# 

**Published:** 1896-07-18

**Authors:** 


**Notices of Births, Deaths, and Marriages** are inserted in this column at a charge of 2s. 6d. for an announcement not exceeding four lines.

**Notice to Advertisers and Subscribers.**

All Advertisements, Orders for Copies of The Hospital and Business Communications should be addressed to **THE MANAGER, (Not to The Editor)**, The Hospital, 428, Strand, London, W.C.

The Cost of Subscription, post free, is as follows:—Per week, 2½d; per quarter, 2s. 8d.; per half-year, 5s. 3d.; per annum, 10s. 6d. Foreign:—Per Annum, 15s. 2d.; payable, in all cases, in advance.

Just Published.

**"Burdett's Hospitals and Charities, 1896."**

Seventh year of publication. Over 1,000 pp. Scarlet cloth, gilt lettered, Price 5s. A most complete guide to British, Colonial, Indian, and American Hospitals, Dispensaries, Nursing and Convalescent Institutions, and Asylums. A few complete sets of the preceding six issues of the "Annual" may still be obtained on application to the Publishers, The Scientific Press (Limited), 428, Strand, London, W.C.

### SCRAPS AND GLEANINGS.

Rochester is one of the latest victims of the street collection craze.

The removal of the Totnes Cottage Hospital to a new site is under consideration.

The board of the Bridgwater General Hospital have decided to admit diphtheria patients to one of its unused wards.

Nearly £94 was raised at Grantham recently on Hospital Saturday, this sum being exclusive of the country district collection.

The Abingdon Cottage Hospital treated 128 patients in 1895, of whom 61 resided in Abingdon, and 67 in the surrounding villages.

The balance-sheet of the Tenby Cottage Hospital shows that the income exceeded the expenditure by a small amount, the figures being, income, £173; expenditure, £170.

During 1895 462 in-patients, 3,389 out-patients, and 2,770 casualties were treated at the Walsall and District Cottage Hospital. The average stay of each in-patient was 16 days.

The Glasgow Hospital for Skin Diseases sent 98 patients for treatment as in-patients at the Western Infirmary at Glasgow, and 1,553 outpatients were treated at the institution itself.

Three Hundred and Seventeen new patients were treated in 1895 at the Glasgow Hospital for Diseases Peculiar to Women. The income amounted to £418, and the expenditure to £378.

The little hospital for crippled children at Bristol, now called the Orthopœdic Hospital and Home for Crippled Children, had 31 patients pass through its wards during the year just ended. Of these 10 were cured.

In the new operating theatre at the Rotunda Hospital, Dublin, the spectators are separated from that part of the theatre in which operations are performed by a large plate-glass screen, the object being to "preserve the air from contamination, and to prevent draughts which might contain organic germs."

The St. Michael's Home for Men and Women can hardly be termed an accurate name for the seaside home of the London Diocesan Deaconess Institution, seeing that it has no accommodation at present for men. For want of about £2,500 the sisters have been only able to complete the centre block, giving room for 16 women patients.

The income of the Stockport Infirmary shows a steady increase during the four years ended December 31st, 1895, in nearly every item. Excluding extraordinary income, the receipts have been £3,766 in 1892, £3,619 in 1893, £3,942 in 1894, and £4,163 in 1895. Extraordinary income has amounted to £127 in 1892, £511 in 1893, £200 in 1894, and £1,110 in 1895.

The average number of days that each in-patient was resident at the Dundee Royal Infirmary during the year 1895-6 was 27, or two less than in the previous year, 2,344 in, 7,680 out, 5,306 district patients, and 1,236 accident cases were treated during the year, and 829 patients were admitted at the convalescent branch of the infirmary at Barnhill.

The attempt made by the present Mayor of Brighton to raise £5,000 in aid of the Sussex County Hospital has been successful, and rather more than that sum has been handed over to the hospital authorities. We are pleased to note that only £55 was spent for printing, postage, and clerical assistance, or very little over one per cent. of the amount raised.

The following figures, with reference to the street collections in connection with Hospital Saturday at Walsall, show that in that town the objection to this form of begging is as pronounced as it is in London. The first street collection was made in 1892 and realized £304 13s. Since then the amounts have been: 1893, £274 8s.; 1894, £241 9s. 6d.; and 1895, £229 13s. 7d.

The first hospital established in Glasgow, with the exception of one for lepers which existed in the fourteenth century, was the Town's Hospital, erected in 1733. This was followed by the Royal Infirmary founded in 1794, at which, until 1871, all kinds of infectious diseases except small-pox were treated. The total number of beds provided at the present time in the Glasgow hospitals is as follows: Charitable hospitals, 1,776; parochial, 1,048; municipal, 902.

The daily average number of beds occupied at the Seacombe Cottage Hospital during 1895 was over fifteen, out of a total of seventeen beds and four cots for children provided. This hospital, which was erected some twenty-five years ago, is now too small for the needs of the inhabitants of the district it serves, but although a site has been provided by one of the original founders, not one-half of the £8,000 required to build a new hospital with forty beds is yet forthcoming.

The total cost of the Ormskirk Cottage Hospital, which was opened this year, was £1,295. It has now been decided, instead of charging the in-patients 7s. a week from the date of their admission, to give all patients admitted with a subscriber's recommendation one month's treatment free, and for any further treatment to make a weekly charge according to means. Patients may now also be admitted without a recommendation on payment from the date of admission of a weekly sum not exceeding two guineas.

Out of a total income of £686 the Retford Cottage Hospital received only £184 in annual subscriptions during the year ended May 31st, 1896. That is to say, annual subscriptions amounted to rather under 27 per cent. of the total receipts, or more than 9 per cent. less than the average receipts from this source, as shown by the analysis of the accounts of 172 cottage hospitals in various parts of Great Britain, given in *Cottage Hospitals*. One hundred and four in-patients, of whom 28 were children, were treated during the year, compared with 79 in 1894-5 and 43 in 1893-4. Eight hundred and four patients were treated at the dispensary, and 1,717 visits paid by the external nurse.

The committee appointed to examine into the charges of mismanagement and extravagance brought against the authorities of the Dawlish Cottage Hospital have exonerated the management from blame, and reported that in their opinion the hospital was suffering "from a lack of subscribers and patients," and that his want could be best remedied "by ascertaining and meeting the wishes of those residents and medical men who at present hold aloof from it; at least so far as those wishes are not very objectionable to the present subscribers and to the medical men now on the staff." The committee found that about fifty former subscribers, who still resided in the town, had ceased to contribute, and of the four medical men who had been on the hospital staff two had withdrawn from it, although still practising in the town.

### The Student of Medicine.

### APPOINTMENTS.

G. F. Aldous, M.R.C.S.Eng., L.R.C.P. Lond., appointed Assistant Surgeon to the South Devon and East Cornwall Hospital. F. R. B. Bishopp, M.A., M.D.Cantab, appointed Hororary Consulting Physician to the Tonbridge Union and Infirmary. R. D. Booth, M.B., C.M.Edin., appointed Junior House Surgeon to the Kimberley Hospital. W. Bradshaw, L.R.C.P.Edin., L.F.P.S.Glas., appointed Medical Officer for the Third District of the Wycombe Union. W. Brown, F.R.C.S.Edin., appointed Honorary Assistant Surgeon to the Cumberland Infirmary, Carlisle. R. E. Burges, M.D., appointed Medical Officer of Health to the Hoole Urban District Council. R. J. Bnrns, M.R.C.S., L.R.C.P. Lond., appointed Medical Officer for the Bishopwearmouth East District of the Sunderland Union. L. V. Cargill, F.R.C.S., appointed Assistant Surgeon to the Royal Eye Hospital. Southwark. D. Crowe, M.B.Dub., B.Ch., appointed Medical Officer for the Wolvey District of the Nuneaton Union. H. Davis, M.R.C.S.Eng., appointed Anæsthetist to the Dental Hospital of London, Leicester Square. D. M. Ffrench, L.R.C.P.I., L.M., appointed Medical Officer for the Mendelsham District of the Hartismere Union. F. J. Fletcher, M.R.C.S., appointed Medical Officer for the Burton Coggles District of the Grantham Out-Relief Union. A. Foxwell, M.D., F.R.C.P., appointed Examiner in Medicine in Cambridge University. W. L. Fry, M.A., B.M., B.Ch., St. John's College, Oxford, late House Physician, appointed House-Surgeon to the Radcliffe, Infirmary, Oxford. W. S. Griffith, M.A., M.B.Cantab., F.R.C.S.Eng., appointed Senior House-Surgeon to the Kimberley Hospital. C. Highet, M.B. and C.M.Glas., appointed Assistant Medical Officer of the Govan District Asylum at Hawkhead, near Paisley. H. Hilliard, L.R.C.P.Lond., M.R.C.S.Eng., appointed Assistant Anæsthist to the Dental Hospital of London. A. Macqueen. M.D.Edin., appointed Medical Officer of Health to the Drayton Rural Sanitary District. J. Mair, M.B., C.M., appointed Resident Medical Officer to the Dundee Poorhouses. G. J. K. Martyn, B.A., M.B., B.C.Cantab., appointed Assistant Resident Medical Officer to the Queen Charlotte's Lying-in Hospital. J. Murray, F.R.C.S., appointed Assistant-Surgeon to Middlesex Hospital. G. R. Murray, M.A., M.D.Camb., M.R.C.P.Lond., appointed Physician to the Royal Infirmary, Newcastle-on-Tyne. J. B. Okill, appointed Surgeon to Her Majesty's Prison at Nottingham. J. P. Parkinson, M.D., M.R.C.P.Lond., appointed Physician to Out-patients to the North-Eastern Hospital for Children, Hackney. H. C. Pearson, M.B., C.M.Edin., appointed Junior House Surgeon to the Borough Hospital, Birkenhead. G. H. Philipson, M.D.Cantab., F.R.C.P.Lond., appointed Consulting Physician to the Royal Infirmary, Newcastle-upon-Tyne. S. Phillips, M.D.Lond., F.R.C.P., appointed Physician to In-patients, St. Mary's Hospital. F. G. Proudfoot, M.A.St.And., M.B., C.M.Edin., late House Surgeon, appointed House Physician to the Radcliffe Infirmary, Oxford. M. Richards, M.D.Lond., appointed Medical Officer of Health to the Chesterfield Town Council. H. Roscoe, M.R.C.S., L.R.C.P.Lond., appointed Assistant Medical Officer to the Crumpsall Workhouse and the New Bridge Street Workhouse of the Township of Manchester. R. A. St. Leger, M.B, C.M., appointed Medical Superintendent Watford and District Isolation Hospitals, District Medical Officer for the Watford District of the Watford Union, and Medical Officer of Health to the Watford Urban District. Mr. E. J. Smyth, appointed Assistant Medical Officer to the Wandsworth and Clapham Union Infirmary. Mary D. Sturge, M.D.Lond., appointed Anæsthetist to the Birmingham and Midland Hospital for Women. W. W. H. Tate, M.D.Lond., M.R.C.P., appointed Assistant Obstetric Physician to St. Thomas's Hospital. R. Taylor, M.R.C.S.Eng., L.S.A., appointed Medical Officer for the Gray's Inn Road Workhouse of the Parish of St. Marylebone. W. J. Taylor, L.R.C.P.Lond., M.R.C.S.Eng., appointed Medical Officer of Health to the Ampthill Urban District Council. B. Wainman, L.R.C.P.Lond., M.R.C.S.Eng., appointed Medical Officer to the Workhouse of the Hunslet Union. F. W. Waters, M.R.C.S., L.R.C.P., appointed Medical Officer for the Berriew District of the Forden Union.

### THE STUDENT OF MEDICINE—*continued.*

### VACANCIES.

The following vacancies are announced this week, the amount of salary in each case being given after the name of the institution:—

**Resident Medical Officers.**

Great Northern Central Hospital, Holloway Road, N.—House Surgeon. Salary £60 per annum, with board, lodging, and laundry in the hospital. Junior House Surgeon. Appointment for six months, with board, lodging, and laundry provided in the hospital. Applications on forms of application provided to be sent to the Secretary by July 20th.

Hospital for Consumption and Diseases of the Chest, Brompton.—Resident House Physician. Applications to the Secretary, by July 21st.

Isle of Wight Asylum.—Assistant Medical Officer, doubly qualified. Salary £100 per annum, with board, lodging, &c., but without alcohol. Applications to the Medical Superintendent, Whitecroft, Newport, Isle of Wight, by July 20th.

Joint Counties Asylum, Abergavenny.—Junior Assistant Medical Officer. Salary £100 per annum, increasing by two yearly instalments of £25 to £150, with board, lodging, and washing. Applications to the Medical Superintendent by July 18th.

London Homœopathic Hospital, Great Ormond Street, W.C.—Resident Medical Officer. Appointment for 12 months, six months as House Surgeon and six months as House Physician. Salary £80 per annum and board and apartments. Application to G. A. Cross, Secretary-Superintendent, by September 15th.

Royal Albert Hospital, Devonport.—Assistant House Surgeon. No salary; board, lodging, and washing provided. Applications to the Secretary of the Medical Committee not later than the first post on July 18th.

Royal Eye Hospital or Royal South London Ophthalmic Hospital, Southwark.—House-Surgeon; will be required to take up residence about October 1st. Salary £50 per annum, with board and lodging. Applications to the Secretary by August 1st.

West Riding Asylum, Menston, near Leeds.—Fourth Assistant Medical Officer. Salary to commence at £100, rising £10 annually to £150, with board and apartments. Applications to the Medical Superintendent by July 20th.

**Non-Resident Medical Officers.**

Glasgow Samaritan Hospital for Women, Victoria Road, Glasgow.—Clinical Assistant (Lady). Applications to Thos. Macquaker, Secretary, 89, West Regent Street, Glasgow, by July 25th.

London Homœopathic Hospital, Great Ormond Street, W.C.—Assistant-Ophthalmic Surgeon. Applications to G. A. Cross, Secretary-Superintendent, by October 1st.

London Throat Hospital, 204, Great Portland Street, W.—Junior Assistant Surgeon. Applications to the Secretary of the Medical Committee by July 18th.

North-West London Hospital, Kentish Town Road, N.W.—Assistant Resident Medical Officer. Also Assistant Surgeon (must be F.R.C.S.Eng., and reside within three miles of the hospital). Applications to Alfred Craske, Secretary, for the former by July 21st, and for the latter by July 20th.

Plymouth Public Dispensary.—Second Medical Officer of the Provident Department, doubly qualified. Appointment for one year, but eligible for re-election. Physician's Assistant, doubly qualified. Salary £60 per annum. Appointment for one year, but eligible for re-election. Applications to the Hon. Secretary, W. H. Prance, 7, Athenæum Terrace, Plymouth, by July 21st.

(393) G. A. CROSS, Secretary-Superintendent.

**LONDON HOMŒOPATHIC HOSPITAL,**

GREAT ORMOND STREET, BLOOMSBURY, W.C.

The Board of Management are prepared to APPOINT an ASSISTANT OPHTHALMIC SURGEON.

Every candidate must be legally qualified, and be or become, on election, a member of the British Homœopathic Society.

Applications, with copies of testimonials, to be sent to the Secretary-Superintendent not later than October 1st.

(394) G. A. CROSS, Secretary-Superintendent.

**SUSSEX COUNTY HOSPITAL**

